# Angiotensin II receptor blockers decreased blood glucose levels: a longitudinal survey using data from electronic medical records

**DOI:** 10.1186/1475-2840-6-26

**Published:** 2007-09-29

**Authors:** Noboru Kitamura, Yasuo Takahashi, Shuukoh Yamadate, Satoshi Asai

**Affiliations:** 1 Division of Hematology and Rheumatology, Department of Medicine, Nihon University School of Medicine, Tokyo, Japan; 2 Division of Genomic Epidemiology and Clinical Trials, Advanced Medical Research Center, Nihon University School of Medicine, Tokyo, Japan; 3 Clinical Laboratory, Nihon University Nerima-Hikarigaoka Hospital, Tokyo, Japan

## Abstract

**Background:**

A beneficial effect on glucose metabolism is reported with angiotensin receptor blocker (ARB) treatment of hypertension. The effect on blood glucose level during the course of treatment with ARBs in clinical cases is uncertain. Our objectives were to survey the changes in glucose and HbA1c levels in patients with hypertension over a one-year period, and to study the correlations between these values and the time after the start of ARB therapy.

**Methods:**

We conducted a retrospective longitudinal survey of blood glucose and HbA1c measurements in Japanese patients aged ≥20 years with newly diagnosed hypertension but without diabetes, who had received ARB monotherapy with candesartan cilexetil, losartan potassium, olmesartan medoxomil, telmisartan, or valsartan during the period from December 2004 to November 2005. Data including 2465 measurements of non-fasting blood glucose in 485 patients and 457 measurements of HbA1c in 155 patients were obtained from electronic medical records of Nihon University School of Medicine. Linear mixed effects models were used to analyze the relationship between these longitudinal data of blood examinations and covariates of patient age, sex, medication, and duration of ARB therapy.

**Results:**

Casual blood glucose level was associated with the duration of treatment (P < 0.0001), but not with age, sex, or medication. Blood glucose level was significantly decreased during the periods of 0~3 months (P < 0.0001) and 3~6 months (P = 0.0081) compared with baseline, but was not significantly different between 6~12 months and baseline. There was no association between HbA1c level and covariates of sex, age, medication and duration of treatment.

**Conclusion:**

Our findings provide new clinical evidence that the effects of ARBs on glucose metabolism may change during the course of treatment, suggesting a blood glucose-lowering effect in the short-term after the start of treatment.

## Background

Clinically, there are many cases in which abnormal glucose metabolism and hypertension appear together. Knowing the effects of blood pressure-lowering agents on blood glucose is important for the treatment of hypertensive patients with abnormalities of glucose metabolism or diabetes. The agents most often used for hypertension today include angiotensin receptor blockers (ARB) and angiotensin converting enzyme (ACE) inhibitors, which are both considered to improve glucose metabolism [1,2]. In addition, much attention has been paid to a prospective clinical trial that showed that ACE inhibitors suppressed the new onset of diabetes [3]. A subsequent report showed that ARBs also suppressed the new onset of diabetes [4]. The magnitude of this effect was around 20–25%. Recent animal experiments have suggested improvement of insulin resistance by ARBs [5,6]. Clarification of the mechanism of this effect is in progress.

In patients with essential hypertension, plasma insulin and blood glucose levels rise together, indicating reduced insulin sensitivity. Because of this, even without the onset of diabetes, a latent rise in blood glucose level may be seen. We thus selected the study subjects from among those not diagnosed with diabetes at the time of ARB administration, and who were being treated with an ARB alone, at a hospital affiliated to Nihon University School of Medicine, using medical record data for patients who had been diagnosed with hypertension and had received an ARB. We examined the changes in blood glucose and HbA1c levels in these patients over a one-year period, and then studied the correlations between these values and the duration of ARB therapy.

## Methods

### Study Population

**Figure 1:**
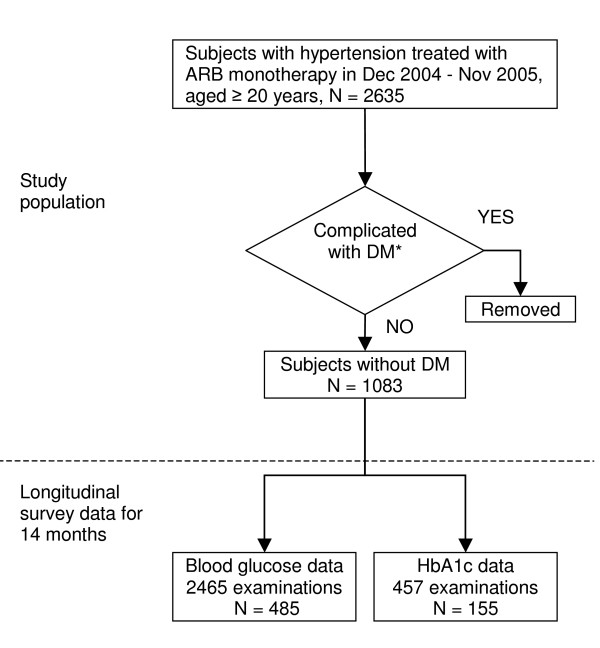
**Flow of study population**. Medical record reviews for longitudinal survey data were conducted for 14 months; 2 months before and 12 months during ARB treatment. Detailed criteria for exclusion are described in the Methods.

The data for this analysis were collected from a clinical database that integrates information of inpatient and outpatient medical records at a hospital affiliated to Nihon University School of Medicine. The study population consisted of 2635 Japanese men and women aged 20 years or older who had been treated initially by ARB monotherapy at usual doses for at least 4 weeks during the period from December 2004 to November 2005, as shown in Figure 1. Exclusion criteria included treatment with insulin and/or a previous diagnosis of diabetes mellitus according to the Committee for the Classification and Diagnosis of Diabetes Mellitus of the Japan Diabetes Society, defined as fasting plasma glucose level ≥126 mg/dl, casual plasma glucose level ≥200 mg/dl, plasma glucose 2 h after 75 g glucose load ≥200 mg/dl, or hemoglobin A1c (HbA1c) level ≥6.5% [7]. Some patients had received other antihypertensive drugs such as ACE inhibitors or calcium channel blockers during the 2 months before ARB therapy and were excluded from our study. The remaining 1083 patients were subjected to further statistical analysis. The ARBs used are listed in Table 1. Clinical data from the study subjects included sex, age at starting treatment, names of drugs, starting date of treatment, duration of treatment, results of non-fasting blood examination including blood glucose and HbA1c levels, which were determined at the time of routine clinical visits, and date of examination. The blood examination data were collected under the criteria of having at least one value during the 2 months before treatment or the duration of treatment up to a maximum of one year. A total of 485 patients from this population were eligible for the study of blood glucose, and 155 subjects for HbA1c.

**Table 1:** Angiotensin II receptor blockers

Generic name	Drug name (Trade name)
candesartan cilexetil	Blopress
losartan potassium	Nu-Lotan
olmesartan medoxomil	Olmetec
telmisartan	Micardis
valsartan	Diovan

### Statistical Analysis

Our main explanatory variables included sex, age at starting treatment, medication, "duration" defined as the timing of measurement in days since the start of treatment, coded as 0, 1, 2, or 3, with 0 indicating "baseline", i.e., ≤ time 0, and 1, 2, and 3 indicating ">0, ≤3 months", ">3, ≤6 months", and ">6, ≤12 months" since the start of treatment, respectively. Our main response variables were repeated measurements of blood glucose and HbA1c in blood examination before and after treatment with antihypertensive drugs. These data were inherently unbalanced because the number and timing of the repeated measurements were different among individuals. Therefore, linear mixed effects models were used to analyze the relationship between these longitudinal data of blood examination and all other covariates [8]. We fitted random intercept and slope models to the data, including age, sex and medication as fixed effect covariates, and duration of treatment as random effect covariates. A "compound symmetry" covariance was used to covary the random intercept and slopes and estimate the covariance between them. We selected the Kenward-Roger method to compute the denominator degrees of freedom for the tests of fixed effects. A multiple-comparison test (Dunnett-Hsu post-hoc analysis) was used to compare the differences in means between "baseline" as a reference and other times. A result was considered statistically significant if the P value was less than 0.05. All statistical analysis was performed with SAS 9.1.3 (SAS Institute Inc., Cary, NC) statistical software.

## Results

### Characteristics of study sample

Of the study population of 2635 patients with hypertension who had been treated with ARB monotherapy, a total of 1083 patients without diabetes were subjected to statistical analysis. Blood examination data are shown in Table 2. Approximately 60% of patients were female and 40% were male, for both casual blood glucose and HbA1c measurements. Approximately 65% of patients were aged over 60 years. The numbers of blood examinations were 2465 and 457 for blood glucose and HbA1c, with 5.08 and 2.95 measurements per subject, respectively.

**Table 2:** Frequency distribution of blood examination data

	Items	
	
Variables	Glucose	HbA1c
PATIENT INFORMATION		
Number of subjects	485	155
Sex		
Female	187 (38.6)	54 (34.8)
Male	298 (61.4)	101 (65.2)
Age, years		
20–49	67 (13.8)	20 (12.9)
50–59	103 (21.2)	33 (21.3)
60–69	150 (31.0)	47 (30.3)
70<	165 (34.0)	55 (35.5)
Medication		
candesartan cilexetil	177 (36.5)	51 (32.9)
losartan potassium	78 (16.1)	35 (22.6)
olmesartan medoxomil	56 (11.5)	21 (13.5)
telmisartan	56 (11.5)	18 (11.6)
valsartan	118 (24.3)	30 (19.4)
DATA INFORMATION		
Number of examinations	2464	457
Treatment duration		
0 = "Baseline"	733 (29.8)	150 (32.8)
1 = "~3 M"	1068 (43.3)	132 (28.9)
2 = "3~6 M"	377 (15.3)	89 (19.5)
3 = "6~12 M"	286 (11.6)	86 (18.8)

Abbreviations: HbA1c, hemoglobin A1c. All data are count (percentage).

### Relationship of covariates to blood glucose and HbA1c

Table 3 presents the results from linear mixed effect models fitted to casual blood glucose and HbA1c data. There was no association between HbA1c level and any of the covariates of sex, age, medication and "duration of treatment". In contrast to HbA1c, the duration of treatment was associated with blood glucose level. However, sex, age, and medication were not associated with blood glucose level. Our study showed no significant differences in blood glucose and HbA1c levels among the different ARB agents during the time course of ARB therapy. Dunnett's multiple-comparison test showed that blood glucose level was significantly decreased in the periods of "0~3 months" and "3~6 months" compared with "baseline", but was not significantly different between "6~12 months" and "baseline". The interaction of duration of treatment and other covariates was not significant (data not shown). This indicates that the effect of treatment duration does not depend on the level of other covariates. Therefore, the tests for individual effects are valid, showing a significant effect of "treatment duration", but no significant effect of sex, age, and medication. These results indicate that the duration of ARB therapy has an influence on blood glucose level, suggesting a blood glucose-lowering effect of ARBs, at least within 6 months of the start of treatment.

**Table 3:** Relationship of covariates to blood glucose and HbA1c measurements

	Glucose (mg/dl)			HbA1c (%)		
		
Variables	LS mean ± SE	(95% CI)	P value	LS mean ± SE	(95% CI)	P value
Sex			0.2268			0.8062
Female	107.80 ± 1.34	(105.15, 110.44)		5.51 ± 0.10	(5.31, 5.71)	
Male	109.70 ± 1.07	(107.58, 111.81)		5.48 ± 0.07	(5.33, 5.64)	
Age, years			0.075			0.3041
20–49	105.09 ± 2.10	(100.96, 109.22)		5.33 ± 0.15	(5.01, 5.64)	
50–59	108.83 ± 1.75	(105.38, 112.27)		5.44 ± 0.13	(5.17, 5.71)	
60–69	109.69 ± 1.43	(106.88, 112.51)		5.62 ± 0.10	(5.42, 5.83)	
70<	111.37 ± 1.32	(108.77, 113.97)		5.61 ± 0.09	(5.42, 5.79)	
Medication			0.4579			0.6887
candesartan cilexetil	110.93 ± 1.31	(108.34, 113.51)		5.62 ± 0.10	(5.41, 5.82)	
losartan potassium	107.73 ± 2.01	(103.77, 111.69)		5.52 ± 0.12	(5.28, 5.77)	
olmesartan medoxomil	106.99 ± 2.24	(102.58, 111.40)		5.37 ± 0.15	(5.06, 5.68)	
telmisartan	108.57 ± 2.16	(104.31, 112.83)		5.43 ± 0.16	(5.10, 5.76)	
valsartan	109.51 ± 1.56	(106.44, 112.59)		5.55 ± 0.12	(5.30, 5.81)	
Treatment duration			<.0001			0.6014
0 = "Baseline"	111.98 ± 1.16	(109.70, 114.27)	reference*	5.51 ± 0.07	(5.37, 5.66)	reference*
1 = "~3 M"	105.86 ± 1.13	(103.64, 108.09)	<.0001*	5.50 ± 0.07	(5.35, 5.65)	0.8209*
2 = "3~6 M"	107.78 ± 1.48	(104.87, 110.69)	0.0081*	5.44 ± 0.08	(5.28, 5.61)	0.2582*
3 = "6~12 M"	109.36 ± 1.63	(106.16, 112.56)	0.1308*	5.54 ± 0.08	(5.37, 5.70)	0.7457*

Abbreviations: HbA1c, hemoglobin A1c; LS means, least squares means; SE, standard error; CI, confidence interval.

*Dunnett-Hsu post-hoc analysis.

## Discussion

Various opinions exist regarding whether reduction of insulin sensitivity can induce hypertension. Regarding the responsible mechanism, the compensatory high plasma insulin accompanying a reduction of insulin sensitivity is said to: 1) increase sympathetic nerve activity [9], 2) promote renal sodium reabsorption [10], 3) induce the growth of vascular smooth muscle [11], and have other similar effects that induce or aggravate hypertension. On the other hand, hypertension itself may cause sympathicotonia and trigger hyperinsulinemia. Furthermore, each may aggravate the other, producing a vicious cycle of hypertension and high blood glucose.

In the present study, a significant reduction in casual blood glucose level was observed with ARB administration. Regarding the mechanism, the general understanding is that after insulin binds to the insulin receptor, tyrosine phosphorylation inside the cell causes activation of insulin receptor substrate-1 (IRS-1) and phosphatidylinositol 3-kinase (PI3-kinase) [12]. As a result, the rate of glucose uptake into the cell increases. Although insulin resistance itself is still poorly understood, there is evidence that the levels of expression of IRS-3 and PI-3 kinase are reduced, although there may be many other genetic abnormalities involved. Folli et al. showed that, in vascular smooth muscle cells, angiotensin II reduces tyrosine phosphorylation of IRS-1 by approximately 50% and reduces binding of IRS-1 to PI3-kinase by 30–50% [13]. ARB and ACE inhibitors block the inhibitory effect of angiotensin II on insulin signal transmission [3,4]. As a result, insulin resistance is improved, and there appears to be a beneficial effect on glucose metabolism. ARBs and ACE inhibitors are thought to have the same actions. In the present study, experiments were conducted using ACE inhibitors as well, but the same trends as for ARBs were noted, and no statistically significant difference was observed. The differences between the two could possibly be due to differences in sample size. In addition, ARBs block the effects of angiotensin II at the receptor level, more so than ACE inhibitors. ARBs may thus be more selective in their effects on the renin-angiotensin system.

The effects of ARBs on glucose metabolism have been reported in several experiments on animals and cell lines. Schupp et al. reported that ARBs induce activation of PPAR γ in cultured adipose cell lines of mice, which partially removes insulin resistance [14]. In addition, Fujimoto et al. showed with the same cell line that telmisartan administration increased the uptake of glucose and expression of GLUT4 protein [15]. Furthermore, Furuhashi et al. showed that blocking of the renin-angiotensin system by an ARB increased the plasma adiponectin level [16]. This is hypothesized to be the mechanism by which ARBs reduce blood glucose. However, even in the case of animal experiments, few reports are available concerning how long ARBs affect glucose metabolism. Among clinical trials as well, there are only a few trials, over short time periods and with small numbers of cases.

A particular feature of this study is that it involved a retrospective survey, which included examination of the changes in blood glucose level over time in 485 hypertensive patients with no glucose metabolism abnormalities, spanning 12 months of ARB therapy. In the present study, we observed no statistically significant difference in results among the different ARBs. In addition, our study showed no significant difference in HbA1c level during the time course of ARB therapy. However, patients with diabetes were excluded in the present study, and this may have been the reason for the lack of significant difference in HbA1c level.

Within the first 3 months of ARB therapy, a significant decrease in blood glucose level was noted. This decrease was maintained for 6 months, though blood glucose level subsequently returned to the same level as at the start of administration, and at 12 months, no significant difference from the baseline level was noted. Since blood pressure was controlled from the start of administration, the initial reduction in blood glucose level appears to have been an effect of ARBs and/or the reduction of blood pressure. However, our observation that blood glucose level tended to increase again after 6 months, together with previous reports that thiazide diuretics and β-blockers reduce insulin sensitivity [17-19], suggest that the initial reduction in blood glucose appears to have been an effect of ARB treatment.

## Limitations

Further study on whether the blood glucose-lowering effect by suppression of the renin-angiotensin system was direct or through some other factor is needed. Although there have been reports that calcium channel blockers and α-blockers improve insulin resistance and glucose metabolism abnormalities, this was not examined in the present study. We are looking into these issues as future subjects of research.

## Conclusion

The duration of ARB therapy was significantly associated with casual glucose level in nondiabetic adults with hypertension regardless of treatment with candesartan cilexetil, losartan potassium, olmesartan medoxomil, telmisartan, or valsartan. Our findings not only support the notion that ARBs can improve glucose metabolism via blocking the inhibitory effect of angiotensin II on insulin signal transmission as suggested elsewhere, but also demonstrate that the effects of ARBs on glucose metabolism are associated with the course of treatment, suggesting a blood glucose-lowering effect within 6 months of the start of ARB therapy.

## Competing interests

The author(s) declare that they have no competing interests.

## Authors' contributions

SA and NK conceived the study and participated in its design. YT performed the statistical analysis. NK and YT drafted the manuscript and interpreted the data. All authors have read and approved the final manuscript.
